# Single-cell RNA sequencing in juvenile idiopathic arthritis

**DOI:** 10.1016/j.gendis.2023.04.014

**Published:** 2023-05-24

**Authors:** Xiwen Luo, Xuemei Tang

**Affiliations:** a Department of Rheumatology and Immunology, Children’s Hospital of Chongqing Medical University, Chongqing 400014, China; b Chongqing Key Laboratory of Child Infection and Immunity, Ministry of Education Key Laboratory of Child Development and Disorders, National Clinical Research Center for Child Health and Disorders, China International Science and Technology Cooperation Base of Child Development and Critical Disorders, Children's Hospital of Chongqing Medical University, Chongqing 400014, China

**Keywords:** Juvenile idiopathic arthritis, Macrophage, Macrophage activation syndrome, Monocyte, Single-cell RNA sequencing, Synoviocyte, T cell

## Abstract

Juvenile idiopathic arthritis (JIA) is one of the most common chronic inflammatory rheumatic diseases in children, with onset before age 16 and lasting for more than 6 weeks. JIA is a highly heterogeneous condition with various consequences for health and quality of life. For some JIA patients, early detection and intervention remain challenging. As a result, further investigation of the complex and unknown mechanisms underlying JIA is required. Advances in technology now allow us to describe the biological heterogeneity and function of individual cell populations in JIA. Through this review, we hope to provide novel ideas and potential targets for the diagnosis and treatment of JIA by summarizing the current findings of single-cell RNA sequencing studies and understanding how the major cell subsets drive JIA pathogenesis.

## Introduction

Juvenile idiopathic arthritis (JIA) is a type of chronic rheumatic arthritis in children. According to a 2014 systematic review, the incidence of JIA ranges from 1.6 to 23 cases per 100,000 people and the prevalence from 3.8 to 400 cases per 100,000 people; overall, JIA incidence and prevalence vary by sex, region, and disease subtype.^1^^,^^2^ JIA is categorized by the International League Against Rheumatism (ILAR) into seven subtypes: systemic JIA (sJIA), oligoarticular JIA (oJIA), RF-positive polyarticular JIA (RF^+^ pJIA), RF-negative polyarticular JIA (RF^−^ pJIA), enthesitis-related arthritis (ERA), psoriasis arthritis (PsA), and undifferentiated arthritis.^3^ JIA is thought to be influenced by genetic susceptibility, the environment, and immune system mechanisms.^4^ Multiple organs can be affected. In particular, joint inflammation can even lead to joint deformity, poor prognosis, and a lower quality of life. The diagnosis of sJIA is based on clinical characteristics after distinguishing it from infectious disease, other inflammatory diseases, or malignancy. It is unique among other childhood chronic arthritis subtypes due to its systemic characteristics with extra-articular features, including a quotidian fever of at least 2 weeks duration, transient rash, hepatosplenomegaly, lymphadenopathy, or serositis.^4^

The treatment of JIA begins with nonsteroidal anti-inflammatory drugs (NSAIDs) and/or intra-articular corticosteroid injections, with a second line of traditional synthetic disease-modifying anti-rheumatic drugs (tsDMARDs), or biological DMARDs. Some patients respond unsatisfactorily to therapy and may develop severe complications, including uveitis, pulmonary lesions, and macrophage activation syndrome (MAS).^5^ Therefore, a comprehensive understanding of the factors influencing disease activity is crucial to earlier identification and treatment. Studies have demonstrated that monocytes or macrophages, natural killer (NK) cells, dendritic cells, neutrophils, and T lymphocytes play distinct roles in JIA, highlighting the role of cell-mediated mechanisms that regulate immune responses in JIA.^6^, ^7^, ^8^, ^9^, ^10^ However, the question of how different cell types contribute to disease progression remains unanswered.

Traditional transcriptome sequencing measures the average expression of individual genes across a large cell population and can be used to investigate differential expression among tissues. It is, however, insufficient for analyzing more diverse cellular systems, and much of the low-abundance information is lost in the overall characterization. Single-cell RNA sequencing (scRNA-seq) has evolved into a favorable technique for high-throughput transcriptome sequencing. It can present the expression profile at the individual cell level, allowing us to address intercellular heterogeneity more effectively, identify new and rare cell types, and gain insight into the regulation of expression mechanisms during cell growth. Utilizing scRNA-seq in the study of JIA can reveal disease heterogeneity, provide new ideas for elucidating the function of specific cell types, and guide treatment methods.

Here, we attempt to summarize the findings from scRNA-seq studies on JIA, examine the challenges, and discuss the potential applications of combining scRNA-seq with multiomics technologies.

### Single-cell RNA sequencing

Traditionally, cells can be defined according to cell morphology or specific expression patterns of certain functional proteins.^11^^,^^12^ The advantage of proteomics techniques is the ability to analyze the final functional products of gene expression, and at the single-cell level, they appear to be limited to a restrictive, preselected library of molecules, which compromises an accurate and comprehensive description of cell phenotypes. Moreover, the transcriptome is an essential component in investigating and maintaining the identity of cells and the biological processes of organisms. Methods used to determine gene expression in individual cells provide another way to classify cells.^13^^,^^14^

Due to intrinsic stochastic processes and external factors, homogeneous cell populations may exhibit considerable heterogeneity in expression patterns. Neighboring cells that share the same microenvironment can express the same transcript at different levels, and this stochasticity leads to transcriptional noise, which is a random, abrupt fluctuation in gene expression that is important in a cell's fate.^15^ However, the advancement of single-cell sequencing technology has made it possible to precisely define cells.

**Figure 1:**
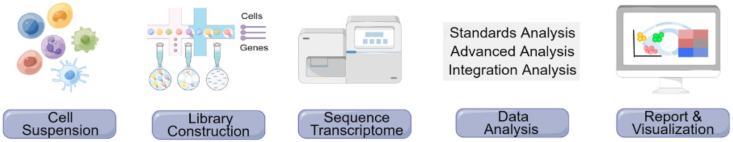
Flow chart of single-cell transcriptome sequencing. Its process focuses on examining isolated cells for quality control, library construction, data analysis, and visualization to reveal the characteristics of cell populations and functions. The figure was drawn by utilizing Figdraw software.

The first report on single-cell transcriptome sequencing by Tang et al was in 2009. Increasingly sensitive and precise single-cell transcriptome sequencing technologies have developed over the past few years, including Quartz-Seq, CEL-seq, MARS-seq, Drop-seq, Smart-Seq, and inDrop.^16^ New platforms reduce costs and enhance information output. Moreover, the big picture can be depicted from the sequencing information. The sequencing program goes through (i) quality control to exclude damaged and dying cells, (ii) normalization to correct the difference in expression of useful reads among cell subpopulations, (iii) batch effect correction, (iv) imputation and smoothing, (v) cell cycle assignment, (vi) feature selection to recognize genes with the strongest biosignature relative to technical noise, (vii) dimensionality reduction and visualization with principal component analysis (PCA) and uniform manifold approximation and projection (UMAP), (viii) unsupervised clustering, (ix) pseudotime to construct transformation pathways in cellular space, and (x) differential expression of a single gene compared to combining datasets (Fig. 1).^17^

### Single-cell RNA sequencing in juvenile idiopathic arthritis

#### ScRNA-seq explored monocytes and bone marrow macrophages in systemic juvenile idiopathic arthritis with macrophage activation syndrome (sJIA-MAS)

The crucial pathophysiology in sJIA, identified as a unique autoinflammatory disease, is the persistent activation of intrinsic immunity and secretion of pro-inflammatory cytokines by monocytes, macrophages, neutrophils, and T cells, resulting in the appearance of systemic clinical symptoms.^7^^,^^18^^,^^19^ Immune cells generate pro- or anti-inflammatory cytokines to maintain innate immunity, particularly by increasing the proliferation of monocytes and macrophages. The rich polarization patterns of monocytes or macrophages in sJIA are also regulated by various noncoding RNAs, making the nuances of cell subtypes difficult to dissect.^18^^,^^20^

Human monocytes are typically divided into three groups: classical, intermediate, and nonclassical populations.^21^, ^22^, ^23^ Traditionally, there are two main phenotypes of macrophages: M1 (classically activated) macrophages and M2 (alternatively activated) macrophages.^18^ Moreover, inflammatory monocytes and tissue-resident macrophages regulate tissue repair and injury, reactive oxygen species (ROS) production, phagocytosis, antigen presentation, apoptosis, and transendothelial migration and differentiation.^23^, ^25^, ^24^

Bulk RNA sequencing of peripheral blood (PB) mononuclear cells (PBMCs) demonstrated that monocytes in sJIA exhibit altered transcriptional activity as an active phenotype. Interestingly, no significant difference between the monocytes of JIA patients with active disease and those with clinically inactive disease (CID) was detected.^26^ Analysis of differential gene expression showed that sJIA patients with elevated monocyte ferritin levels, using 210 ng/mL as a ferritin cutoff point, exhibited activation of pro-inflammatory transcription. Schulert et al found the highest enrichment for the M2 phenotype and relatively low enrichment for the M1 phenotype.^9^^,^^26^ These results also proved that macrophages in sJIA may have mixed polarization phenotypes.

Furthermore, the up-regulation of IFN-γ and tripartite pattern-containing 8 (TRIM8) was observed in sJIA patients, and the overexpression of TRIM8 is a specific manifestation of sJIA.^26^ Schulert and colleagues applied scRNA-seq to test macrophages in bone marrow (BM) from one patient with sJIA-MAS and ultimately identified 11 subpopulations, including an enriched IFN-responsive cell population. Again, TRIM8 was a marker gene of the smaller subgroup from two different BM macrophage populations with a significantly altered transcriptional profile. The population marked by TRIM8 displayed a strong IFN-γ-induced signature, with significant up-regulation of genetic pathways, including responses to innate immunity and cytokines, and a large, activated network of transcription factors.^26^ In particular, the pathways involved in intracellular granule movement were significantly up-regulated in this macrophage population, including the MAS-associated gene *STXBP2* (syntaxin binding protein 2), verifying the correct identification of hemophagocytic macrophages.^26^^,^^27^ The robust *IFN-γ*-induced macrophage profile is consistent with the demonstration that IFN-γ alone can interact directly with macrophages, inducing hemophagocytosis and leading to inflammatory desmoplastic anemia. ScRNA-seq enables researchers to reacquaint the cell types with their functional states more thoroughly.^26^

However, the link between IFN-γ and TRIM8 is not clear. Previous studies have elucidated that TRIM8 is involved in cell proliferation, cancer, immunity, and inflammation. It directly targets the *TP53* gene, induces TP53-dependent cell cycle arrest, and exerts antiproliferative effects as a tumor suppressor. TRIM8 can also function as an oncogenic protein that leads to cell proliferation by cooperating with nuclear factor κB (NF-κB) and STAT3.^28^^,^^29^ Furthermore, TRIM8-mediated stabilization of XIAP (X-linked Inhibitor of Apoptosis Protein), an important regulator of cell death and autophagy, prevents the activation of caspase-3 and resists apoptosis.^30^ The function of TRIM8 may primarily depend on three pathways: the p53 oncogenic signaling pathway, the NF-κB pathway, and the JAK-STAT pathway, specifically STAT3 (signal transducer and activator of transcription 3) and its microenvironment. TRIM8 is involved in conflicting biological processes and exerts regulatory, antiproliferative, anticancer, and pro-inflammatory effects.^31^^,^^32^ Whether TRIM8 has a link with IL-18 and IFN-γ through the signaling pathways mentioned above remains to be discovered.

Comprehensive analysis of high-dimensional data reveals complex immune alterations in inflammatory diseases. Investigators have identified genes associated with specific cytokine environments and activated leukocyte subsets. Moreover, sJIA patients showed dysregulated responses to TLR4, TLR8, and TLR7 stimulation during disease remission and cessation of treatment. Isolated monocytes from sJIA were low in IL-1 inhibitor aryl hydrocarbon receptor (AHR) expression at baseline and accumulated higher levels of intracellular IL-1β following stimulation.^26^ The aryl hydrocarbon receptor (AHR) is a ligand-activated transcription factor that triggers a broad immune response, such as the regulation of pro-inflammatory cytokine production by monocytes and macrophages.^33^ AHR down-regulation can cause monocytes to differentiate toward macrophages, and *in vitro* assays demonstrated differentiation more toward macrophages rather than the dendritic cell phenotype. This process may increase the incidence of MAS in these patients. The overexpression of *AIP* and *AHRR* encoding AHR repressor leads to the down-regulation of the AHR pathway and transition to MAS, supported by the work of Cepika et al.^34^

High expression of AHRR (AHR repressor) and HIF-1α inhibits AHR signaling in Th17 cells and regulatory T cells (Tregs) in autoimmune hepatitis (AIH). The imbalance between Tregs and Th17 cells is linked to a low level of CD39, which is associated with the dysfunction of the AHR pathway that results from aberrant inhibition or nonclassical activation binding to Erα.^35^ Unlike the abnormally elevated expression of AHR found in synovial tissues from patients with rheumatoid arthritis, overactivation of the AHR pathway regulates cytokine expression, including growth factors, which may promote inflammation and bone destruction mediated by activated macrophages, osteoclasts, dendritic cells, and suppressed osteoblasts.^36^, ^37^, ^38^ These findings suggest a shift in cell expression profiles across tissues and disease states and involve the interaction between innate and adaptive immunity. Overall, the blockade of these inhibitory AHR pathways may be a potential therapeutic approach.^39^

**Figure 2:**
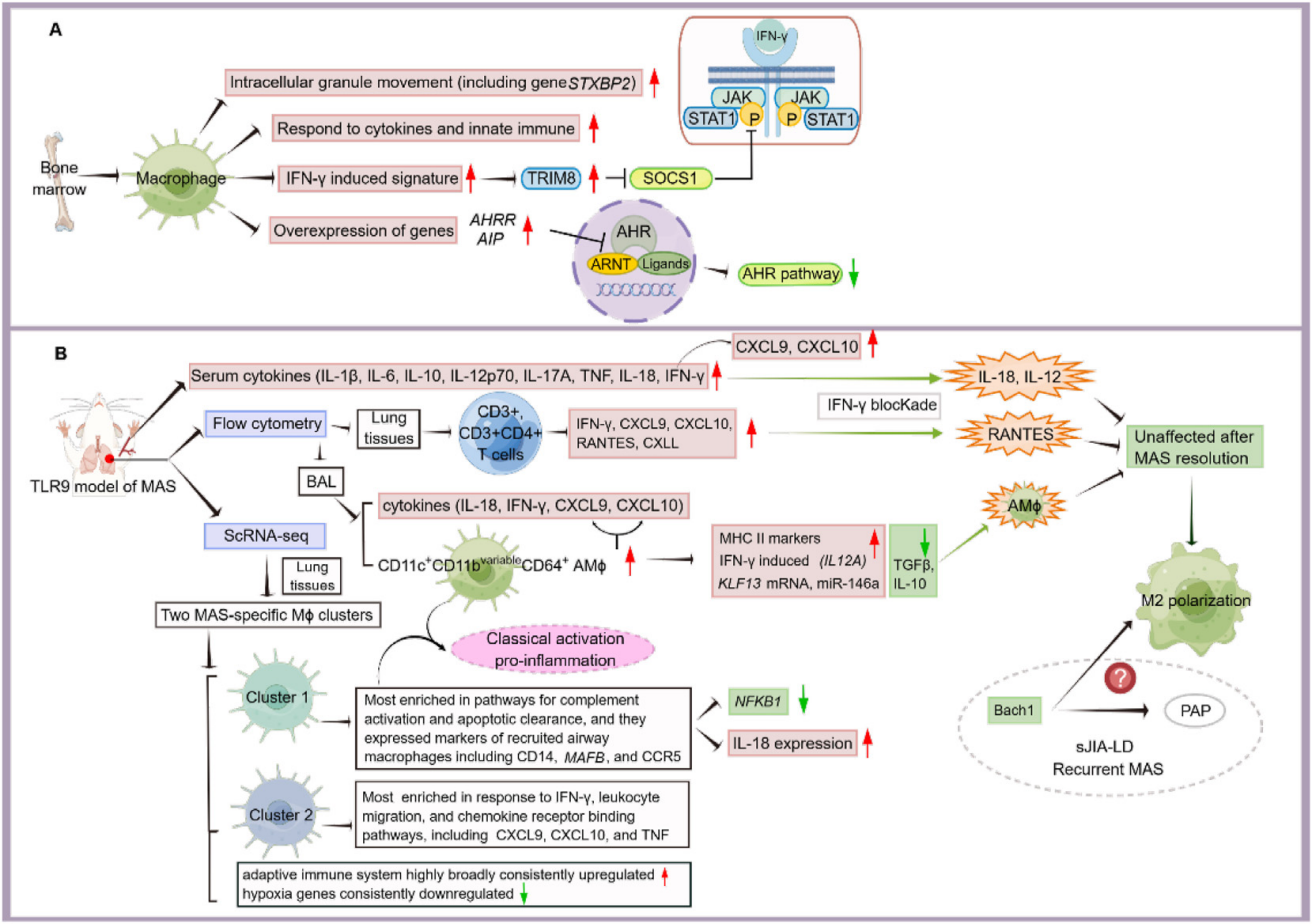
Identification of monocytes or macrophages in sJIA patients and a mouse model of MAS by using flow cytometry combined with scRNA-seq. **(A)** The mechanisms associated with bone marrow-derived macrophages in a patient with sJIA-MAS. IFN-γ induction possibly regulates the JAK-STAT1 signaling pathway via TRIM8. AMϕ expressed markers are related to intracellular granule movement, cytokine response, and intrinsic immune response. The overexpression of the AHRR and AIP genes may down-regulate the AHR pathway, which is associated with MAS transition **(B)** The immune response in blood monocytes and AMϕ in a mouse model of MAS induced by TLR9. The acute MAS model mice showed high expression of inflammatory factors in serum, lung tissues, and lavage fluid. AMϕs were mainly classically activated. Flow cytometry identified a unique AMϕ phenotype in BAL fluid, namely, CD11c^+^ CD11b^variable^ CD64^+^ AMϕs. ScRNA-seq identified two distinct subpopulations of MAS AMϕ in lung tissues and showed extensive high expression of adaptive immunity and persistent low expression of hypoxic genes. The acute MAS model mice exhibited M2 polarization after remission, but IL-18, IL-12, and RANTES expression was barely affected, suggesting an incomplete effect of IFN-γ blockers. In recurrent MAS model mice, reprogrammed AMϕ toward classic polarization activated inflammatory responses. The red arrows represent up-regulation. The green arrows represent down-regulation. A short straight line indicates an inhibitory or blocking effect. A tapering arrow at the end indicates remission-related features. The figure was drawn by utilizing Figdraw software. AHR, aryl hydrocarbon receptor; *AHRR*, aryl hydrocarbon receptor repressor; *AIP*, aryl hydrocarbon receptor interacting protein; AMϕ, alveolar macrophage; ARNT, aryl hydrocarbon receptor nuclear translocator; BAL, bronchoalveolar lavage; CCR5, C–C motif chemokine receptor 5; CXCL9, C-X-C motif chemokine ligand 9; CXCL10, C-X-C motif chemokine ligand 10; IL-1β, interleukin-1β; IL-6, interleukin-6; IL-10, interleukin-10; IL-12, interleukin-12; IL-12p70, interleukin-12p70; IL-17, interleukin-17; IL-18, interleukin-18; IFN-γ, interferon-gamma; JAK, Janus kinase; *KLF13*, Kruppel-like factor 13; *MAFB*, MAF BZIP transcription factor B; MHC II, major histocompatibility complex II; *NFKB1*, nuclear factor Kappa B subunit 1; P, phosphorylation; *STXBP2*, syntaxin binding protein 2; *STAT1*, signal transducer and activator of transcription 1; RANTES, regulated upon activation, normal T-cell expressed and presumably secreted; SOCS1, suppressor of cytokine signaling 1; TGFβ, transforming growth factor-beta; TLR9, Toll-like receptor 9; TNF, tumor necrosis factor; TRIM8, tripartite motif containing 8.

Together, monocytes in sJIA possess both anti-inflammatory and pro-inflammatory properties. Approximately 10%–15% of sJIA patients progress to MAS.^26^^,^^40^ Moreover, IFN-γ has been increasingly considered a vital driver of MAS. TRIM8 overexpression was found to be a feature that distinguished pro-inflammatory macrophages with IFN-γ response characteristics from other macrophages. In line with animal models of MAS, several findings indicate that IFN-γ activation is followed by a rapid flow of inflammatory monocytes to tissues. In addition, IL-18 contributes to IFN-γ production and enhanced responses to IFN-γ (caused in part by an increase in TRIM8), both of which may be possible pathophysiological characteristics underlying the high incidence of MAS in sJIA. TRIM8 may serve as a promising treatment target for acute flares of cytokine storms, including MAS, and for patients with prolonged sJIA who are at risk for recurrent MAS. More research is needed to assess the mechanism by which TRIM8 affects IFN-γ expression (Fig. 2A).^26^ The transcriptome changes that lead to MAS remain to be elucidated, and identifying the dynamic cell types that trigger MAS during patient follow-up is essential.

#### Inspiration for the treatment of sJIA-related lung disease (sJIA-LD) from scRNA-seq exploration of pathological changes in MAS lung disease model mice

SJIA combined with pulmonary lesions is a disease that has been gradually recognized in recent years. Common pulmonary complications include pleurisy or pleural effusion. In the last decade, concerns about lung pathologies have increased.^41^ Chronic lung diseases have emerged as a new cause of death in children with sJIA-MAS. Previous studies have noted the relationship among alveolar macrophages in sJIA, MAS, and sJIA-LD.^41^^,^^42^ Specifically, Gao et al used a TLR9 MAS mouse model to study the pathogenesis of lung disease. They found that the mouse lungs exhibited mild but diffuse CD4-dominated perivascular interstitial inflammation with elevated alveolar macrophages (AMϕ). AMϕ expressed IFN-γ, IFN-γ-inducible genes, and IFN-inducible chemokines in the acute phase. Consistent with cytokine storms, single-cell transcriptome sequencing confirmed IFN-driven AMϕ transformation. Mice with acute MAS showed increased serum expression of IL-10, IL-12p70, IL-17A, IL-1β, IL-6, IL-18, IFN-γ, TNF, and the IFN-induced chemokines CXCL9 and CXCL10. Mouse lung tissues showed a significant increase in the levels of IFN-γ, CXCL9, CXCL10, RANTES, and CXCL1. It is worth noting that IL-1β, IL-6, and IL-10 levels did not change in lung tissues.^42^

IL-18, IFN-γ, CXCL9, and CXCL10 levels were significantly increased in the bronchoalveolar lavage fluid (BAL) of MAS model mice in the acute phase, similar to those of sJIA-LD patients. In contrast, there were few changes in other cytokines levels, such as IL-1β, IL-6, IL-10, IL-12, and TNF. AMϕs showed significant up-regulation of IFN-γ-induced pro-inflammatory genes (including *IL12A* and *CXCL9*) but no changes in other polarization markers (*e.g.*, *HMOX1* and *TGFB*). There was also an increase in pro-inflammatory miR-146a, similar to monocytes in children with sJIA and some patients with rheumatoid arthritis (RA) or psoriatic arthritis (PsA).^42^, ^43^, ^44^ MiR-146a is believed to exert a crucial role in the “fine-tuning” of the monocyte NF-κB signaling pathway, capable of reducing NF-κB and IRF3 activity.^45^^,^^46^ However, there is controversy about whether miR-146a promotes M1 or M2 phenotype polarization.^47^ In addition, a marked elevation of the transcription factor Kruppel-like factor *(KLF)-*13 mRNA is essential for M1 polarization in mice. Interestingly, the key regulatory miR-125a-5p, which directly targets *KLF13*, was not significantly altered in alveolar macrophages in a TLR9-induced MAS mouse model.^42^ MiR-125 can target *KLF13*, suppress M1 activation, and promote M2 polarization in mice. *In vitro*, miR-125a-5p overexpression in macrophages shifted polarization toward the M1 phenotype, similar to what was observed in monocytes of sJIA. Highly elevated miR-125a-5p levels were observed in monocytes from sJIA patients with active disease compared to those with inactive disease and were associated with systemic features.^48^ In contrast, other studies reported that miR-125a could target IRF4 and stimulate M1 but suppress M2 polarization in tumor-associated macrophages.^49^ Later studies pointed out that the binary taxonomy could no longer satisfy the diverse differentiation patterns of macrophages. These contradictory findings may reflect differences between species, tissues, and macrophage types.

Moreover, CD11c^+^ CD11b^variable^ CD64^+^ AMϕ clearly expressed MHC class II surface markers, symbolizing the classical activation pattern, while M2 markers, such as CD206, remained unchanged. The expression of the macrophage surface markers CD11c^−^ CD11b^+^ did not change significantly.^42^ In conclusion, AMϕ exhibits changes in surface MHCII and gene expression during the acute phase of MAS, indicating pro-inflammatory polarization, IFN activation, and secretion of IFN-induced chemokines into the BAL fluid. Notably, AMϕ in MAS model mice had a unique gene expression profile, including reduced expression of *IL12A* or *KLF13* as well as *TGFB* and *IL10*, implying the existence of distinct AMϕ isoforms and a role for IFN-induced AMϕ in MAS lung pathology.^42^

A notable increase in both populations of typical monocytes, inflammatory monocytes, mesenchymal monocytes, and MAS-specific macrophages in lung tissue was also observed. The major surface markers of specific macrophage subpopulation 1 were *Cd5l*, *C1qb*, *C1qc*, *Itga9*, *Mertk*, *Slc40a1*, *Spic*, *Rgl1*, *Nr1h3*, *Dok2*, *etc*. Gene expression was enriched in complement activation, apoptotic clearance pathways, and expressed markers associated with the recruitment of airway macrophages, such as *CD14*, *MAFB*, and *CCR5*, which, unlike classical monocytes, down-regulate *NFKB1* and NF-κB inhibitors and up-regulate IL-18. The major surface markers of another subpopulation were *H2-Aa*, *H2-Abf*, *Sod2*, *Cd74*, *Marcksl1*, *H2-Eb1*, *Fth1*, *ll1m*, *H2-DMb1*, *Sdc4*, *etc*. The genes specific to this subgroup were enriched in the IFN-γ response, lymphocyte migration, and chemokine receptor binding pathways such as *CXCL9*, *CXCL10*, and *TNF*. Most notably, genes associated with the adaptive immune system were highly, extensively, and consistently up-regulated in most cell populations in MAS, while hypoxic genes continued to be down-regulated. Up-regulated genes in classical monocytes were selectively enriched in energy metabolism and TCA genes, and Stat1-centered transcriptional network regulation was projected to be associated with Irf1 and Rel-responsive transcription. Extensive IFN-mediated transcriptional responses were observed in pulmonary lesions associated with MAS, and the expansion of potentially novel monocytes and macrophages was observed.^42^

Studies have also found that different T-cell subsets were enriched. Acute MAS model mice had significantly increased CD3^+^ and CD3^+^ CD4^+^ T lymphocytes, and the T-cell population showed activation of IL-2-mediated signaling. Moreover, a minor but significant reduction in AMϕ and up-regulation of T cells and CD45^−^ epithelial cells was discovered in TLR9 agonist CpG-induced acute phase MAS model mice. Consistent with sJIA patients, serum cytokines such as IFN-γ and IL-6 in these mice returned to normal by the later stages of the disease, but IL-18 remained elevated even when the disease was clinically inactive. In MAS model mice in remission of systemic symptoms, the proportion of total cells and AMϕ in alveolar lavage fluid increased moderately, and the levels of chemokines in alveolar lavage fluid and serum were normal. The levels of the IFN-induced genes *CXCL9* and *IL12A* and M1-related miR-146a also returned to normal levels. There was no significant difference in the down-regulation of *HMOX1* and IFN-activated STAT1 and Bach1, as well as the up-regulation of *TGFB*.^42^ Bach1 is a transcriptional repressor that may be essential for limiting the M2 phenotype, and its deficiency worsens some forms of pulmonary alveolar proteinosis (PAP). Do these results suggest that inappropriately active pro-resolution processes such as Bach1 down-regulation during chronic or recurrent MAS may produce PAP? Several transcription factors of the Kruppel-like factor (KLF) family have also been proposed. A small and significant increase in CD11c^+^ CD11b^variable^ CD64^+^ AMϕ, without significant differences in CD206 expression, has also been described, which up-regulates the STAT6 regulatory cluster in the M2 phenotype.^42^^,^^50^ This study suggests that during MAS remission, genes and transcript expression associated with monocytes and macrophages are altered and involved in regulating inflammation, metabolic pathways toward anti-inflammation, and polarization.

AMϕ in acute MAS has a pro-inflammatory phenotype induced by IFN-γ and transforms into an anti-inflammatory phenotype during disease remission. The remission of MAS is related to an increase in anti-inflammatory type AMϕ and interstitial lymphocyte infiltration. In contrast, recurrent MAS activates alveolar inflammation and reconverts AMϕ to a pro-inflammatory state, leading to lung injury. Blockade of IFN-γ attenuated the expression of cytokines, including IL-6, IL-10, TNF, CXCL9, and CXCL10, but the expression of IL-12 and RANTES was almost unchanged. MHCII activation markers were down-regulated, especially in CD11c^+^ CD11b^variable^ CD64^+^ AMϕs, and *IL-12A* and miR-146a showed little change, possibly reflecting the incomplete effect of monoclonal antibodies on blocking systemic IFN-γ. Mice carrying IFN-γ-insensitive macrophages presented remission of systemic symptoms and pulmonary inflammation in a MAS model. Thus, IFN-γ-driven mechanisms associated with pulmonary inflammation may be a potential new therapeutic direction for sJIA-LD.^42^

The link between MAS and sJIA-LD in a MAS mouse model revealed transcriptional activation of the IFN pathway, suggesting that specific bone marrow-derived macrophage populations in MAS exhibit substantial expansion, which is possibly a signal generated by different cell populations.^42^ Histological pulmonary infiltration in acute MAS model mice is more prominent in remission and is accompanied by a shift of alveolar macrophages from M1 to M2 polarization.^42^ Alveolar macrophages in remission showed more extensive transcriptional changes reflecting alternative activation and pro-resolution phenotypes, including increased *TGFβ* mRNA, activation of STAT6 expression, and reduced expression of STAT1 targets associated with IFN-γ.^51^^,^^52^ However, alveolar macrophages in recurrent MAS model mice reproduced the pro-inflammatory phenotype (Fig. 2B).^42^ Moreover, Bach1 may be involved in chronic or recurrent MAS and lead to PAP if pro-resolution programs are not properly activated.^53^ Similarly, a decrease in monocyte transcription factor (TF) M1-associated IRF5 and M2-associated STAT6 and an increase in only M2-associated PPARG were found after the use of rilonacept. The decrease in pro-inflammatory monocytes after pharmacological inhibition of IL-1 may be associated with a significant reduction in TF IRF5. In addition, PPARG activation suppresses IL-1β production and may be involved in the anti-inflammatory response.^54^ Decreased transcripts of IL1B are related to the lower activity of sJIA achieved by rilonacept and potentially serve as predictive biomarkers for drug response.^55^ The existence of an IRF5^low^, STAT6^low^, and PPARG^high^ phenotype in monocytes, which eventually emerged regardless of whether the treatment response occurred early or late, must be recognized by single-cell sequencing.^50^

Of note, the link between IL-1 or IL-6 antagonists and MAS and sJIA-LD is still uncertain. It is unclear whether exposure to IL-1 or IL-6 antagonists might lead to the development of sJIA-LD. Studies that followed up with patients without IL-1 or IL-6 antagonist treatment still had sJIA-LD.^41^^,^^56^ By analyzing the serum proteomics of sJIA-LD patients, Chen et al found that elevated or decreased proteins in sJIA-LD were not associated with MAS and that sometimes sJIA-LD is not accompanied by MAS manifestations. These findings might imply different origins of pathogenesis in MAS and sJIA-LD. Further studies suggested that up-regulated intracellular adhesion molecule 5 (ICAM-5) could serve as a biomarker for sJIA-LD to distinguish it from other forms of interstitial lung disease.^57^^,^^58^ Overall, more research is urgently needed to determine the pathogenic mechanisms linking immune cell differentiation in SJIA to disease outcome, MAS, and lung disease.

#### ScRNA-seq revealed T-cell polarization in the synovial fluid in oJIA

Studies have shown that arthritic JIA exhibits an imbalance of Tregs, Th1 cells (IFN-γ secreting T cells), and Th17 cells (interleukin-17 secreting T cells) in adaptive immunity.^59^, ^60^, ^61^ Effector T (Teff) cells can be classified into distinct subtypes, including Th1, Th2, and Th17 cells. Many studies have illustrated the presence of a cell subpopulation with a mixed phenotype of Th1 and Th17 cells in JIA patient synovial fluid.^62^^,^^63^ Such differences may reflect patient stratification and suggest that JIA includes different forms of chronic inflammatory arthritis with distinct biological profiles.

Recently, Amélie et al showed the expression of Th1-related markers by CD4^+^, CD8^+^, and γδT cells in the synovial fluid (SF) of oJIA patients, but they did not observe Th17 cell enrichment. Compared with control PB, oJIA joints had higher concentrations of memory CD4^+^, memory CD8^+^, and γδ T cells that expressed Th1 cytokines (IFN-γ) and chemokine receptors (CXCR3) but not IL-17. The most pronounced upregulation of IFN-γ and CXCR3 was observed in SF-derived CD4^+^ memory T (Tmem) cells. However, the authors were unable to identify CXCR3^+^ IFNγ^+^ cells because intracellular cytokine detection requires stimulation that causes CXCR3 down-regulation.^64^

It has been reported that there is an increase in IL-17^+^ CD4^+^ T cells in JIA SF.^65^^,^^66^ In the work by Amélie and colleagues, IFN-γ^+^ IL-17^+^ CD4^+^ Tmem cells were slightly more frequent within the SF than in the PB. CD161 (Th17-associated chemokine receptor) is an alternative marker of Th17 cells. In the SF of oJIA patients, CD161^+^ CD4^+^ Tmem cells were slightly enriched but at a lower frequency than CXCR3^+^ CD4^+^ Tmem cells. IFN-γ^+^ CD161^+^ CD4^+^ Tmem cells were increased in the joint, whereas IL-17^+^ CD161^+^ CD4^+^ Tmem cells were not. Over half of the CXCR3^+^ cells in the joint did not coexpress CD161, suggesting a considerable population of classical Th1 cells. Fergusson and colleagues proposed that CD161 also defines a population of innate-like T cells that produce IFN-γ in response to IL-12 and IL-18. Increased expression of IL-12 and IL-18 receptors was detected in SF CD4^+^ T cells in transcriptomic studies. *KLRB1*, which encodes CD161, was expressed in all effector memory clusters but was remarkably absent in central memory cells.^67^ Overall, a large proportion of CD4^+^ Tmem cells in oJIA SF secreted IFN-γ but not IL-17.

Th1 cells were predominantly found in oJIA SF and associated with disease severity. CD4^+^ Tregs in oJIA SF also expressed Th1 markers. SF Tregs and SF Teffs up-regulated *IFNG*, *CXCR3*, and *TBX21* expression, which encodes the Th1 lineage-defining transcription factor T-bet. Gene enrichment analysis revealed that genes involved in IFN-γ signaling were highly enriched in SF Tregs and Teffs compared to PB. Gene sets related to antigen presentation, T-cell receptor (TCR) signaling, and type I IFNs were also enriched in SF Tregs and SF Teffs. The abundance of Th17-related genes not expressed in SF Tregs was significantly higher than those in PB Tregs. Transcripts of the Th17 chemokine receptor *CCR6* and master gene *RORC* increased slightly in SF Teffs compared to PB Teffs, but there was no difference in IL-17 expression.^64^

Tregs remain stable in oJIA SF.^68^ CXCR3^+^ SF Tregs remained hypomethylated in the conserved noncoding sequence 2 (CNS2) of *FOXP3* and were considered stable Tregs. SF Teffs showed significantly lower methylation levels at the *FOXP3*, *CTLA4*, and *IKZF2* loci. These genome-level studies demonstrated stable epigenetic imprinting of the Treg program, which preserved the inhibitory capacity of CXCR3^+^ and CXCR3^−^ SF Tregs, both of which effectively inhibited SF Teff proliferation.^64^ In contrast to previous studies, Teff in the SF of JIA patients resisted Treg-mediated inhibition.^69^^,^^70^ These inconsistent results may be due to suppression assays that cocultured a population of Tregs with SF Teffs. A small fraction of IFN-γ^+^ SF Tregs detected by flow cytometry may represent an unstable Treg population. Further work is necessary to understand the function of IFN-induced Tregs in oJIA.

Studies have defined Tregs as CD4^+^ CD25^+^ T cells, which may actually contain activated nonregulatory T cells.^69^^,^^71^ Another report elucidated the majority population of SF Tregs (CD4^+^ CD25^+^ CD127^low^ T cells), while Amélie et al focused on CXCR3^+^ Tregs.^70^ ScRNA-seq displayed heterogeneity in Th1-skewed CD4^+^ T cells in oJIA SF. Key genes related to Tregs (*FOXP3*, *IL2RA*, *IKZF2*, *CTLA4*, *TNFRSF18*) and Th1 cells (*CXCR3*, *TBX21*, *STAT1*, *IL12RB2*) were detected in all Treg clusters.^64^^,^^68^ One cluster had the highest expression of the Th1-associated genes *TBX21*, *IL12RB2*, and CXCR3, while the lowest levels of expression were *CCR6* and *KLRB1* and regulatory genes related to anti-inflammatory cytokines. Notably, cells in this cluster also expressed genes found in follicular Tregs related to T-cell–B-cell interactions, including *ICOS*, *PRDM1*, *MAF*, and most markedly, *BATF*.^64^

The single-cell analysis also identified Tph-like Teff cells that were originally described in the rheumatoid arthritis synovium stimulating B-cell responses.^72^ Interestingly, this population had a greater frequency and clonality in antinuclear antibody (ANA)-positive patients.^73^ However, the additional finding indicated that the presence of ANA autoantibodies was not related to variations in the frequency of these T-cell populations.^64^ Fischer et al revealed that clonally expanded CD4^+^ Tph cells accumulated in the joints of ANA-positive JIA patients and promoted CD21^low/−^ CD11c^+^ double-negative B-cell differentiation, which might cause the autoimmune response in the joints of ANA-positive JIA patients.^74^ Hence, the nuanced relationships between T cells and ANA need to be understood. In addition, a population of CD4^+^ T cells expressing multiple cytotoxic markers was found. CD4^+^ cytotoxic T lymphocytes (CTLs) kill cells in an MHC class II-restricted manner.^75^ Moreover, CD4^+^ CTLs have been found most frequently in viral infections or antitumor immunity and appear to be closely associated with Th1 cells.^76^^,^^77^

**Figure 3:**
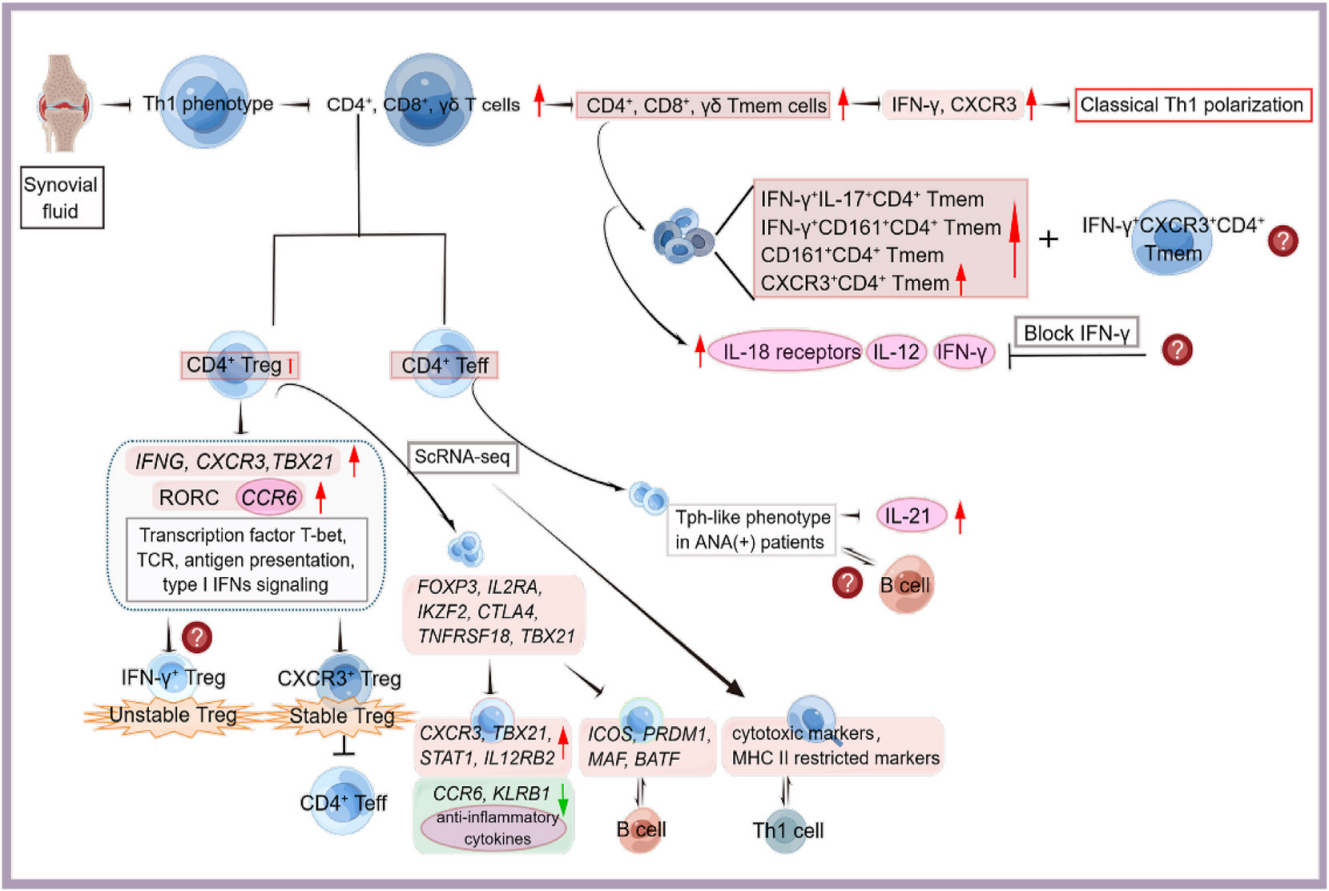
Description of T-cell subpopulations in SF at the single-cell level. The figure demonstrates a predominantly Th1 phenotype in oJIA SF. CD4^+^, CD8^+^, and γδ T cells mainly expressed IFN-γ and CXCR3 but not IL-17, suggesting classic Th1 polarization. Although CD4^+^ Tmem cells expressed the surface markers IL-17 and CD161, these cells did not significantly secrete IL-17. IFN-γ^+^ CXCR3^+^ CD4^+^ Tmem cells cannot be accurately identified due to experimental factors. Single-cell sequencing detected CD4+ Treg cells in SF that also had a Th1 phenotype, but the presence of anti-inflammatory and pro-inflammatory properties suggested heterogeneity of the disease and immune response, while the presence and function of unstable Treg cells were unknown. In addition, a group of Tph-like phenotype cells in CD4^+^ Teff cells may be involved in the disease process in ANA-positive patients, but the relationship between B cells and disease severity is also unknown. The figure was drawn by using Figdraw software. BATF, basic leucine zipper ATF-like transcription factor; *CCR6*, C–C motif chemokine receptor 6; *CTLA4*, cytotoxic T-lymphocyte associated protein 4; CXCR3, C-X-C motif chemokine receptor 3; *FOXP3*, forkhead box P3; *ICOS*, inducible T-cell costimulator; *IFNG*, interferon gamma; *IKZF2*, IKAROS family zinc finger 2; *IL2RA*, interleukin 2 receptor subunit alpha; *IL12RB2*, interleukin 12 receptor subunit beta 2; *KLRB1*, killer cell lectin-like receptor B1; *MAF*, MAF BZIP transcription factor; PMDR1, PR/SET domain 1; *RORC*, RAR related orphan receptor C; SF, synovial fluid; *TBX21*, T-box transcription factor 21; Teff, effector T cell; Tmem, memory T cell; Treg, regulatory T cell; *TNFRSF18*, TNF receptor superfamily member 18.

The diverse groups of SF Teffs and their differentially expressed genes related to Th1, Th17, Tph lineage, T-cell activation and depletion, memory T cells, and CTLs are all involved in the pathogenesis of JIA. Blocking IFNγ to restore immune regulation in JIA may be a potential treatment.^78^ The higher frequency and clonality of the Tph-like population found in ANA-positive patients coincides with the recent observation of an increased frequency of CD4^+^ T cells producing the Tph-associated cytokine IL-21 in ANA-positive JIA patients.^73^ However, the capacity of Tph-like cells in JIA SF to promote B-cell maturation and pathological autoantibody production remains unknown (Fig. 3).

#### Single-cell analysis revealed heterogeneity of juvenile idiopathic arthritis fibroblast-like synoviocytes with implications for disease subtype

Fibroblast-like synoviocytes (FLS) are central to the persistence of JIA inflammation and can express key disease-specific chemokines.^79^ JIA FLS showed dysregulated TGF signaling and a hypertrophic chondrocyte phenotype. These traits, in addition to contributions from the catenin network, may impact the endochondral bone formation and regional growth disturbances in oJIA. Particularly in FLS from oJIA patients, overexpression of bone morphogenetic protein 4 (BMP-4) may be crucial in the pathogenesis of the disease, with repercussions for treatment in the future.^80^ A further study by Simonds et al confirmed previous findings by emphasizing the heterogeneity of FLS and their role in the invasive destruction process of JIA.^81^ They depicted FLS from oJIA, extended-to-be (ETB), and pJIA. Chondrocyte-like cells are the predominant cell subpopulation and are essential for cartilage development. Variable markers were shown in different JIA subtypes, including FBLN1, S100A4, COMP, TIMP1, COL3A1, HAPLN1, and SFRP4 (Table 1). In particular, FLSs exhibit an increasing chondrocyte-like phenotype as the patient’s progress to a polyarticular course, whereas ETB has a distinct genetic fingerprint that can be recognized before progressing to more severe disease courses.^82^

**Table 1:** Heterogeneity in FLS from SF and synovial tissues in oJIA, ETB, and Pjia.

Gene	Comparison group	Roles	
FBLN1	oJIA *vs.* ETB	Related to chondrocyte proliferation	Proteins expressed by chondrocytes promote cartilage development
S100A4	oJIA/ETB *vs.* pJIA	A marker of fibroblasts	
COMP	oJIA *vs.* pJIA	A marker of cartilage turnover	
TIMP1	oJIA *vs.* pJIA	Metalloproteases inhibitor	
COL3A1	ETB *vs.* pJIA	Mutations in COL3A1 related to arthritis and bone disorders	
HAPLN1	ETB *vs.* pJIA	Linked to rheumatic arthritis	
SFRP4	ETB *vs.* pJIA	Inhibiting Wnt signaling	
GREM1, GREM2	oJIA *vs.* ETB	BMP antagonists, participating in chondrocyte differentiation	
TIMP3	ETB *vs.* pJIA	A metalloproteinase inhibitor, a feature of chronic rheumatic inflammatory diseases	
CRLF1	pJIA *vs.* oJIA	Suppression of CRLF1 expression leads to differentiation of MSCs as chondrocytes	
MFAP5	pJIA *vs.* oJIA	Promoting tumor cell proliferation and regulating the ERA/MMP signaling pathways	

*Abbreviations*: BMP, bone morphogenetic protein; COL3A1, collagen type III alpha 1 chain; COMP, cartilage oligomeric matrix protein; CRLF1, cytokine receptor-like factor 1; ETB, extended-to-be; FBLN1, fibulin 1; FLS, fibroblast-like synoviocyte; GREM1, gremlin 1, DAN family BMP antagonist; GREM2, gremlin 2, DAN family BMP antagonist; HAPLN1, hyaluronan and proteoglycan link protein 1; MFAP5, microfibril associated protein 5; MSC, mesenchymal stem cell; S100A4, S100 calcium-binding protein A4; SFRP4, secreted frizzled-related protein 4; SF, synovial fluid; TIMP1, TIMP metallopeptidase inhibitor 1; TIMP3, TIMP metallopeptidase inhibitor 3.

Chondrocyte-like cells have unique genetic embryonic fingerprints that distinguish JIA subtypes despite overlapping subpopulations. lRRC15, GREM1, and GREM2 are overexpressed in chondrocytes of persistent oJIA FLS compared to those in pre-expansion pJIA FLS. Kobayashi et al proposed that GREM1, a specific gene of cancer-associated fibroblasts (CAFs), was involved in bone morphogenetic protein (BMP) signaling. High levels of GREM1 and SLR in patients with colorectal cancer (CRC) were related to diverse survival.^83^ However, Ren et al demonstrated that GREM1 promoted cancer cell invasion in breast cancer. Targeting GREM1 could benefit the treatment of breast cancer patients with high Grem1 expression.^84^ Moreover, persistent GREM1 activity is required to maintain epithelial pancreatic ductal adenocarcinoma (PDAC) subpopulations.^85^ Liu et al revealed that GREM2 inhibited the browning program of visceral preadipocytes by antagonizing the BMP4/7-SMAD1/5/8 signaling pathway.^86^ These results implied that GREM1 and GREM2 could regulate the immune response and fibroblast differentiation through the BMP signaling pathway.

In addition, S100A4, TIMP3, and NBL1 were overexpressed in pre-expansion oJIA FLS compared to pJIA FLS. CRLF1, MFAP5, and TNXB were also overexpressed in persistent oJIA FLS compared to pJIA FLS.^82^ Similarly, fibroblasts with SPINT2^high^, MFAP5^high^, and a few WIF1^high^ were identified in systemic sclerosis-associated interstitial lung disease (SSc-ILD) mesenchymal cells via single-cell analysis, and the evidence suggested that this population actively proliferates.^87^ Therefore, fibroblasts or fibroblast-like cells detected in different tissues or diseases may be potential targets for immunotherapy and prognostic evaluation.

Chondrocyte-like cells are one of the most dominant cell populations in JIA. Chondrocyte-like cells in FLS of pJIA down-regulated the expression of genes that are specific to joint development and collagen formation. ETB JIA appears to have a unique genetic imprint that can be identified before progressing to a more severe state. As the disease progresses toward a polyarticular state, FLS become more chondrocyte-like and less like fibroblasts or smooth muscle cells, which may account for the impaired joint growth observed in JIA.^82^

## Discussion

Current scRNA-seq studies have elucidated a significant number of distinctive monocyte or macrophage populations, T cells, and B cells present in JIA, which might exacerbate the problem of distinguishing the targets that cause the pathological changes in JIA. However, the fine classification of various cell subtypes prompted us to explore various PB mononuclear cell types and potential biological markers at the individual cell level.

Here, we summarize the application of single-cell analysis in distinguishing cellular subpopulations and potential biomarkers in the pathogenesis of JIA. The multilevel information supports the heterogeneity of JIA and suggests more stratification among patients is needed. Deeper mining of single-cell data may help in patient differentiation and precise diagnosis. It may even be integrated with treatment to advance clinical care and drug resistance recognition strategies. However, since JIA patients are subjected to drug use due to severe clinical symptoms early in the disease progression, it is difficult to obtain the pathological background of the disease before treatment and immune alterations after treatment, and there may be unavoidable destruction or loss of effective cells, low coverage, and biased sequencing results. Therefore, the optimization of single-cell sequencing combined with proteomics or other research strategies to identify cell subpopulations and signaling pathways will be beneficial for future multidimensional screening and validation of biomarkers, and accurate typing and early prognosis of JIA and complications.

## Author contributions

X.W.L. drafted the manuscript. X.M.T. contributed to the conception and critically reviewed and edited the manuscript. All authors approved the final manuscript.

## Conflict of interests

The authors declare that the research was conducted in the absence of any commercial or financial relationships that could be construed as potential conflict of interests.

## Funding

This work was supported by grants from the National Key R&D Program of China (No. 2021YFC2702003).

## Review criteria

PubMed was searched for English-language published articles using the terms “juvenile idiopathic arthritis” OR “juvenile chronic arthritis”, and “single-cell RNA sequencing” OR “scRNA-seq”, in combination with, “macrophage activation syndrome”, “lung disease”, “monocyte” OR “macrophage”, “T cell”, “B cell”, “fibroblast-like synoviocyte” OR “chondrocyte-like cell”, and “microRNA”. The reference lists of selected papers were also searched for additional relevant articles.
